# Bibliometric study of neuroinflammation in autism spectrum disorder

**DOI:** 10.3389/fpsyt.2023.1086068

**Published:** 2023-01-19

**Authors:** Yingying Shen, Jiu-Gen Zhong, Wan-Ting Lan, Yin-Hua Li, Jia-Heng Gong, Ben-Xuan Zhao, Xiao-Hui Hou

**Affiliations:** ^1^School of Sport and Health, Guangzhou Sport University, Guangzhou, China; ^2^School of Kinesiology, Shanghai University of Sport, Shanghai, China

**Keywords:** autism spectrum disorder, neuroinflammation, bibliometrics, CiteSpace, VOSviewer

## Abstract

**Background:**

Neuroinflammation is closely associated with the occurrence and development of autism spectrum disorder (ASD). This study aims to describe the global development history and current status of neuroinflammation in ASD from 2004 to 2021 and reveal the research hotspots and frontiers to provide a reference for scholars in related fields to carry out further research.

**Methods:**

Journal articles on ASD and neuroinflammation-related research were obtained from the Web of Science Core Collection (WOSCC) database from its inception to 2021. Literature was analyzed visually by VOSviewer, CiteSpace, and R language, including publication analysis, author, institution, national/regional cooperative network analysis, and keyword analysis. We screened the most accumulatively cited 10 experimental papers in the field and the most cited 10 experimental papers in the last 2 years (2020 and 2021) for combing.

**Results:**

A total of 620 publications were included in this study, and the number of publications has increased in recent years. The United States (256, 41.29%) was the country with the largest number of publications. King Saud University (40, 6.45%) was the most published institution; Laila Al-Ayadhi Yousef was the most published researcher; the *Brain Behavior and Immunity* was the main journal for the study of neuroinflammation in autism, having published 22 related articles. Keyword co-occurrence analysis showed that short chain fatty acid, mast cells, and glial cells have been the focus of recent attention. Burst keywords show that gut microbiota and immune system are the future research trends.

**Conclusion:**

This bibliometric study describes the basic framework for the development in the field of neuroinflammation and ASD through an exploration of key indicators (countries, institutions, journals, authors, and keywords). We found that the key role of neuroinflammation in the development of ASD is attracting more and more researchers’ attention. Future studies can investigate the changes in cytokines and glial cells and their related pathways in ASD neuroinflammation. Immunotherapy to inhibit neuroinflammation may be intensively studied as a direction for ASD treatment or intervention.

## 1. Introduction

Autistic spectrum disorder (ASD), also referred to as autism, is a common, highly heritable, and heterogeneous neurodevelopmental disorder characterized by impairments in social communication and social interaction along with restricted, repetitive behaviors, or interests (1). The current global population prevalence is estimated at 1–2% (2) and has been on the rise (3). The pathogenesis of ASD is still unclear, and there is also a lack of targeted clinical treatment (4). However, with the development of neurobiology in recent years (5, 6), more and more scholars have devoted themselves to the study of related neuroinflammatory mechanisms (7–9). Since Vargas et al. (7) first identified active neuroinflammatory processes in the cerebral cortex, white matter, and cerebellum of ASD patients, many studies have also reported persistent inflammation in different brain regions of ASD patients, mainly manifested as increased elevated pro-inflammatory cytokines in the cerebrospinal fluid, increased brain-specific autoantibodies, and altered immune cell function (10–17). Meanwhile, in the immune system of ASD, there is an imbalance in the number and proportion of immune cells involved in both the innate and adaptive immune responses (18, 19). Abnormalities in these immune cells lead to autoimmune and neuroimmune dysregulation. For example, IL17 signaling in systemic immune cells was found to play an important role in bidirectional brain-peripheral inflammatory communication (20). IL-17 receptor signaling in monocytes may potentiate the effects of IL-17A released by other immune cells and may aggravate neuroinflammation in ASD (21). This evidence reveals that neuroinflammation plays a key role in the pathogenesis of ASD.

Although there were reviews on the topic of neuroinflammation in autism, most of them were about a branch or a specific subfield of neuroinflammation in ASD, and they rarely estimated the current progress and future trend in the field from a macro perspective. By contrast, bibliometrics uses mathematical and statistical methods for quantitative and qualitative analysis of the literature (22), such as co-citation, citation, and co-occurrence studies, which can enable us to objectively understand the history and progress of research in related fields and predict future research directions (23). Citation rankings are often used in the medical field (24, 25) to assist in identifying the peer-reviewed literature with the greatest academic impact (26) and as a basis for ranking journals. To understand the international research focus of ASD and the content of the neuroinflammatory mechanisms, through the collection of relevant literature on the Web of Science database, we analyzed the research hotspots and contents and summarize the main research mechanisms. We used visual analysis software to analyze authors, countries of publication, institutions, journals, and keywords in this field using co-citation and co-linear analysis as the main technical means of bibliometric analysis. We also analyzed research papers with high citation ranking in this field to examine the development history and current status of ASD neuroinflammation and finally revealed the research hotspots. The study provided a reference for research on the pathogenesis and intervention of ASD.

## 2. Methods

### 2.1. Data acquisition and search strategy

The Web of Science Core Collection (WOSCC) database, which includes over 12,000 of the most influential international journals, and is considered the most prominent bibliographic database of peer-reviewed scientific publications on many research topics (27, 28), was used as the data source. We searched the Science Citation Index Expanded (SCI-expanded), Social Sciences Citation Index (SSCI), Conference Proceedings Citation Index-Science (CPCI-S), Emerging Sources Citation Index (ESCI), Conference Proceedings Citation Index—Social Science and Humanities (CPCI-SSH), Arts and Humanities Citation Index (A&HCI), Book Citation Index—Science (BKCI-S), Book Citation Index—Social Sciences and Humanities (BKCI-SSH), Index Chemicus (IC), and Current Chemical Reactions (CCR-EXPANDED) databases. The search strategy was set as follows: (((TS = (Neuroinflammat* OR “neurogenic inflammati*” OR “Nervous Inflammat*” OR “central inflammat*” OR “Neuroinflammation in the central nervous” OR “Brain inflammat*” OR “systemic inflammat*”)) AND TS = (“Autistic Disorder” OR autism OR autistic OR ASD OR “Autism Spectrum Disorder” OR “Asperger Syndrome”))). The search period was from 2004 to 2021, the language type was limited to English, and the document type was set to articles or reviews. Moreover, database search and export were conducted on a single day, March 13, 2022, for the sake of avoiding the possible bias that might come from significant fluctuations in the number of studies as well as citations.

### 2.2. Data analysis

This bibliographic analysis was carried out using VOSviewer (1.6.17) (29), CiteSpace (5.7.R4) (30), and R language (4.2.1) software (31).

Because VOSviewer is a distance-based bibliometric tool focusing on the visualization of bibliometric networks (32), this study uses VOSviewer for country, institution, journal, author collaboration networks, and keyword overlay analysis. The parameters of VOSviewer were set as follows: counting method (full counting) and “ignore documents with a large number of countries/institutions/authors” (maximum number of countries/institutions/authors per document is 25) (33).

CiteSpace enables the dynamic visualization of bibliometric networks evolving (34), so this study used CitesSpace for keyword co-occurrence, clustering, and burst. The parameters of CiteSpace were set as follows: link retaining factor = 2, look back years = –1, e for top *N* = 2, time span = 2004–2021, years per slice = 1, selection criteria = top 50 (35).

The bibliometrix, ggplot2, reshape2, and tidyverse packages in R (36, 37) were used to extract information on keywords, countries and years, and then make heat maps of country publications with times and keyword frequencies. In addition, VOSviewer was used in conjunction with Gephi 0.9 (38) to produce a geographical visualization of country collaboration, and Microsoft Excel 2021 was used for statistics and tabulation.

## 3. Results

### 3.1. Analysis of publication outputs

**FIGURE 1:**
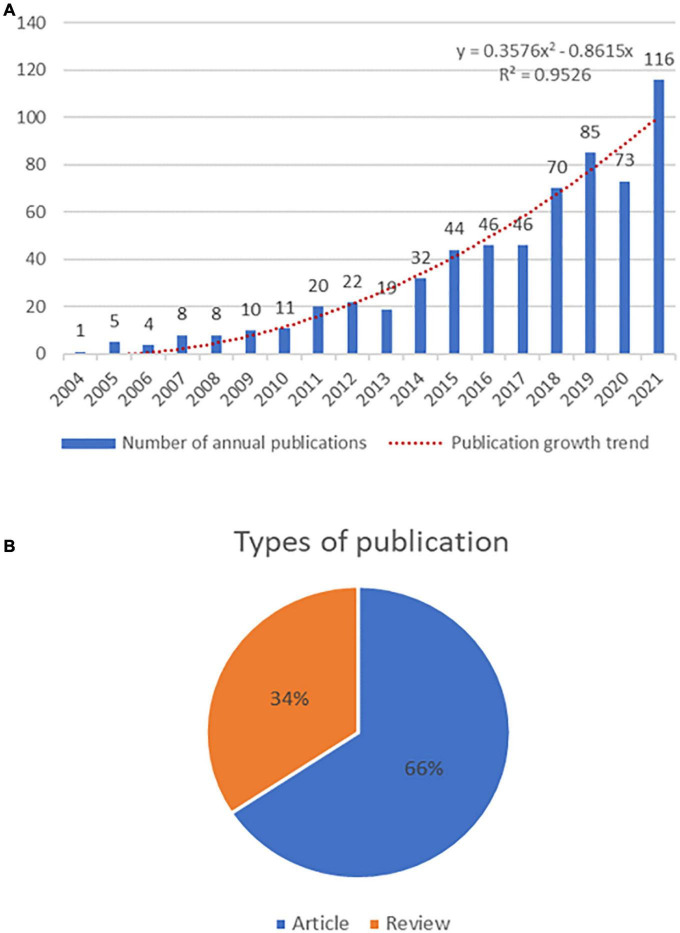
**(A)** The combination chart of the number of annual publications. **(B)** Type of publications.

The annual publication volume is an evaluation index that reflects the degree of attention of the research field. As shown in Figure 1A, a total of 620 papers between 2004 and 2021 met the inclusion criteria. In the initial stage from 2004 to 2013, the average annual number of articles showed an increasing trend, indicating that scholars began to pay attention to this field at this stage. In the second stage from 2013 to 2021, the annual publications showed an overall exponential growth trend, indicating that the number of scholars focusing on the field in this stage is gradually increasing. Furthermore, the generalized additive model was used to evaluate the relationship between the number of papers and the year of publication (39), which showed that the model was very consistent with the annual distribution trend of the literature (*R*^2^ = 0.953). The prediction curve shows that the research literature in this field will continue to increase in the future. Among the types of published articles (Figure 1B), there were 211 review articles, accounting for 34%, and 409 research papers, accounting for 66%, with nearly twice as many research papers as review articles.

### 3.2. Analysis of the most productive countries

**FIGURE 2:**
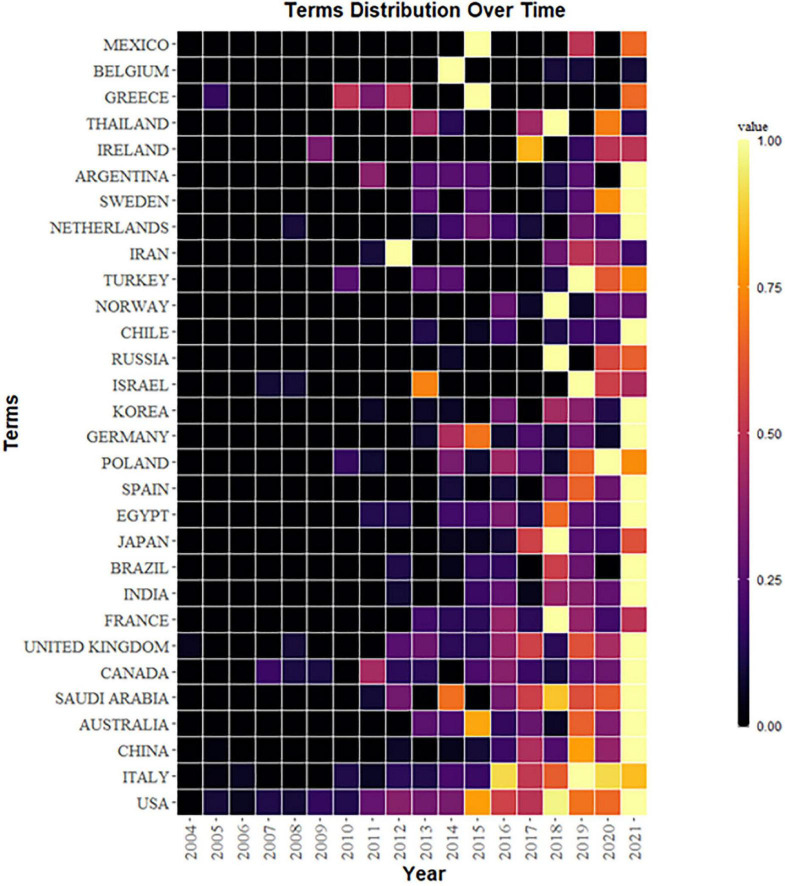
Heat map of country distribution over time: The closer the color is to yellow, the closer the value is to 1, which represents the larger number of publications in the country.

**FIGURE 3:**
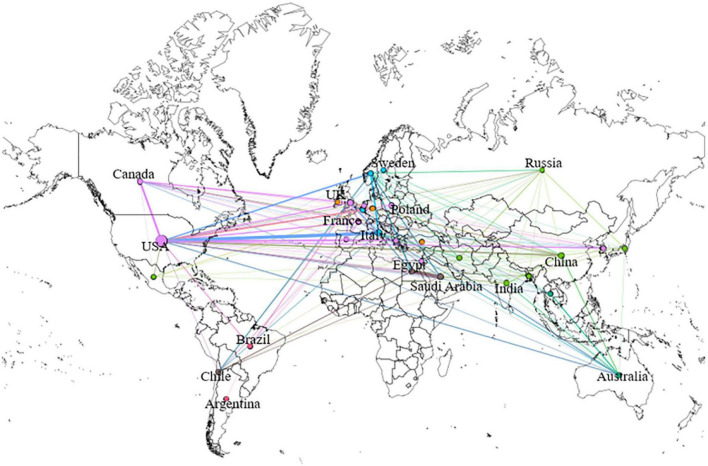
Geography map of national cooperative: The size of the circles shows the number of publications by country. There is a connection between countries, which means that there is cooperation between the two countries. The same color of the wires means that these countries form academic groups.

All publications were produced in 65 different countries. As shown in Table 1, the five most productive countries were the United States, with a total of 256 studies and 13,616 citations, Italy, with 71 studies and 3,911 citations, Saudi Arabia, with 45 studies and 964 citations, China, with 45 studies and 789 citations, and England, with 41 studies and 819 citations. Notably, Canada, which had a small number of publications, had the highest number of citations compared with countries with the same volume of publications, while China and India ranked high in the number of articles published but had lower citations than average. As can be seen from the heat map in Figure 2, the United States was the first country to start and continue to focus on this field. Nearly half of the articles were from the United States, indicating that the United States maintains a high level of attention to ASD neuroinflammation. It is worth noting that since 2016, more countries, such as Italy, Saudi Arabia, and England, have also had ongoing concerns in this field. The geographical map (Figure 3) shows that there is not much cooperation between countries/regions, so cross-regional and cross-country cooperation needs to be strengthened as the research progresses.

**TABLE 1:** Top 10 countries, institutions, journals, and authors.

Country/Region	Publication	Citation	Institution	Publication	Citation	Journal	Publication	Citation	IF	JCR	Author	Publication	Citation
USA	256	13,616	King Saud University	40	894	Brain Behavior and Immunity	22	652	7.22	Q1	Laila Al-Ayadhi	19	677
Italy	71	3,911	Harvard University	34	1,931	Journal of Neuroinflammation	21	1,229	8.32	Q1	Afaf El-Ansary	19	496
Saudi Arabia	45	964	University of California System	32	2,573	International Journal of Molecular Sciences	15	145	5.92	Q2	Theoharis C. Theoharides	17	1,161
China	45	798	Egyptian Knowledge Bank Ekb	30	844	Neuroscience and Biobehavioral Reviews	14	789	8.99	Q1	Sheikh Fayaz Ahmad	11	170
UK	41	819	Harvard Medical School	25	1,512	Frontiers in Cellular Neuroscience	13	464	5.51	Q1	Sabry M. Attia	11	170
India	35	418	Johns Hopkins University	21	2,243	Frontiers in Psychiatry	10	81	4.16	Q3	Saleh A. Bakheet	11	170
Canada	34	2,423	Tufts University	21	1,245	Molecular Autism	10	295	7.51	Q1	Ahmed Nadeem	10	167
Egypt	30	844	Tufts Medical Center	18	1,443	Neurochemistry International	10	196	3.92	Q2	Salvatore Chirumbolo	9	274
Australia	28	1,443	Massachusetts General Hospital	17	967	Autism Research	9	207	5.22	Q1	Mushtaq Ahmad Ansari	8	72
France	22	521	Udice French Research Universities	16	345	Behavioral Brain Research	9	755	3.33	Q3	Derrick F. MacFabe	8	731
						Medical Hypotheses	9	266	4.41	Q2			

### 3.3. Analysis of the most productive institutions

**FIGURE 4:**
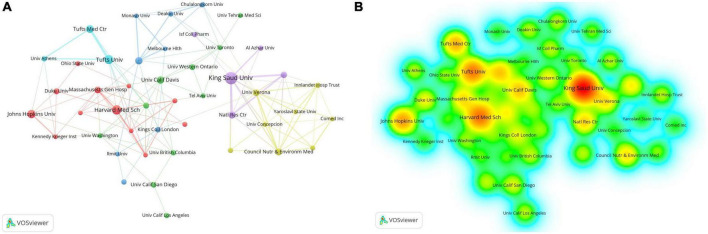
**(A)** The cooperation network visualization map of institutions based on VOSviewer: The thickness of the line represents the strength of cooperation between institutions. **(B)** The density visualization map of institutions based on VOSviewer: The density of color indicates how many publications for institutions.

A total of 329 institutions have made contributions to the published research in neuroinflammation and ASD. Table 1 shows that the top three institutions in terms of article volume, including King Saud University, published 40 articles with 894 citations; Harvard University published 34 articles with 1,931 citations; the University of California System published 32 articles with 2,573 citations. Although Saudi Arabia had the highest number of articles, three of the top five institutions belonged to the United States, which was also consistent with the results of the national ranking of articles. The institutional cooperation network map (Figure 4) shows that the connections among the various institutions are scattered, and there is no close cooperation among them.

### 3.4. Analysis of the higher-impact journals

**FIGURE 5:**
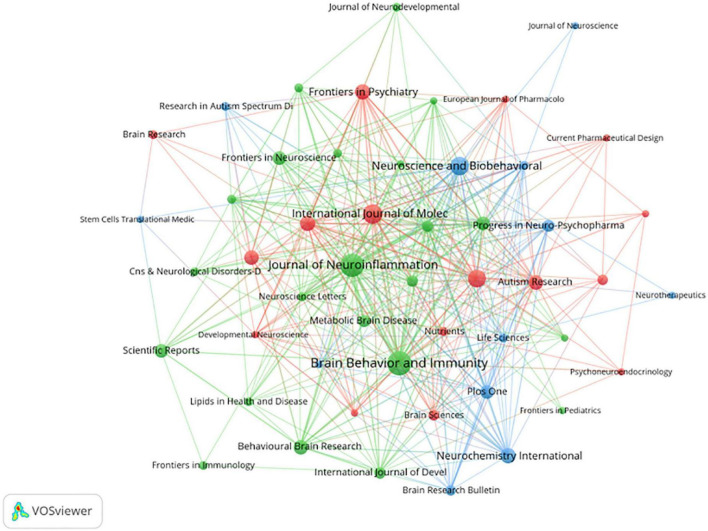
The cooperation network visualization map of journals based on VOSviewer: The thickness of the line represents the strength of cooperation between journals.

Articles on neuroinflammation in ASD were published in 275 journals (Figure 5). As shown in Table 1, the journal with the top three publications was the *Brain Behavior and Immunity*, which published 22 articles with a total of 652 citations, the *Journal of Neuroinflammation*, which published a total of 21 articles and was cited 1,229 times, and *The International Journal of Molecular Sciences*, which published 15 articles with a total of 145 citations. Table 1 shows the top 10 journals with the largest number of publications and their most recent impact factors (IF). In the Journal Division (JCR), 54.5% of journals classified were Q1, and among the publishers of the top 10 journals, five were in the UK, three in Switzerland, two in the US, and one in the Netherlands.

### 3.5. Analysis of the most influential authors

**FIGURE 6:**
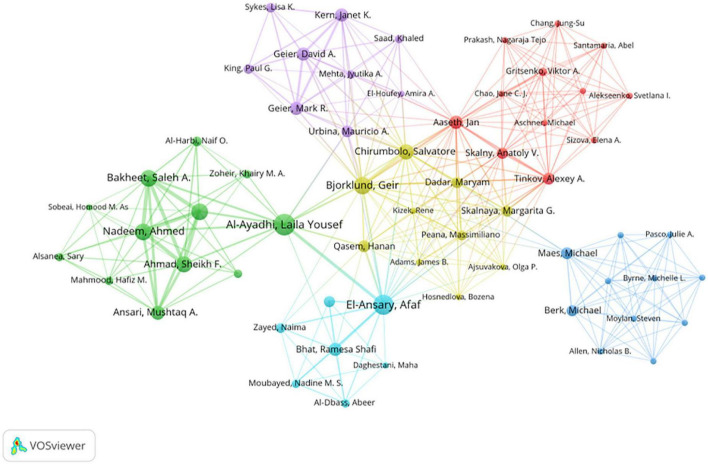
The cooperation network visualization map of authors based on VOSviewer: The node size represents the number of articles published by the author, and the different color blocks represent different academic groups.

A total of 2,584 authors contributed to the field between 2014 and 2021. As shown in Table 1, the top three authors with the most publications were Laila Al-Ayadhi (19 papers), Afaf El-Ansary (19 articles), and Theoharis C. Theoharides (17 articles). Laila Al-Ayadhi and Afaf El-Ansary are both from the Autism Research and Treatment Center at King Saud University, and Theoharis C. Theoharides is from Nova Southeastern University. However, Figure 6 shows the formation of more dispersed clusters among the authors, suggesting that there is a tendency for research teams/labs in the field of ASD neuroinflammation to strengthen collaboration over time.

### 3.6. Analysis of keywords

#### 3.6.1. Keyword co-occurrence

**FIGURE 7:**
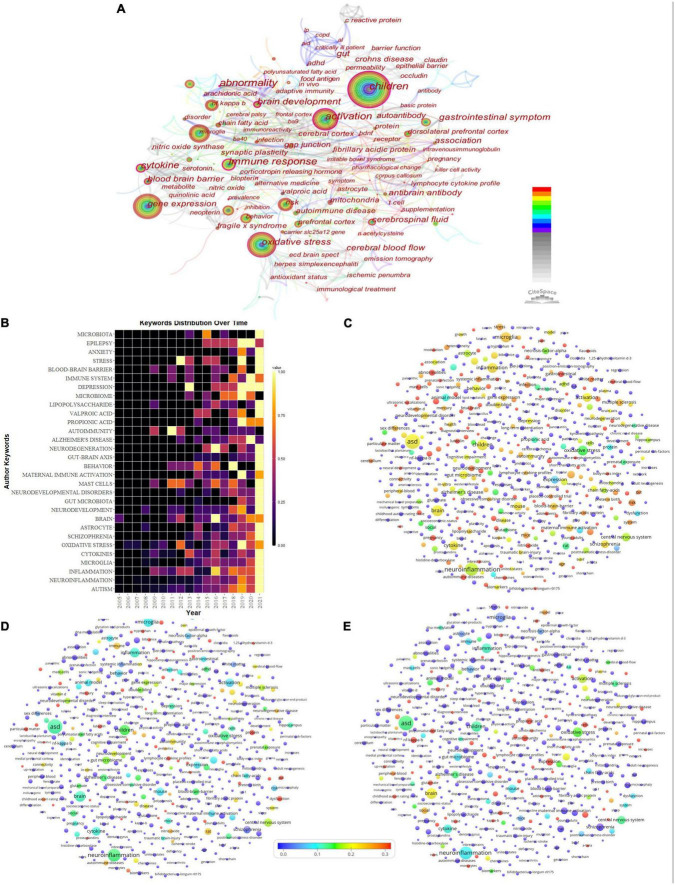
Analysis of the research hotspots. **(A)** Network visualization of author keywords. **(B)** Heat map of top 30 keywords’ frequency over time. **(C)** Map of US keyword frequency superimposed on world keyword frequency. **(D)** Map of Italy keyword frequency superimposed on world keyword frequency. **(E)** Map of Saudi Arabia keyword frequency superimposed on world keyword frequency. Panels **(C–E)** show the contribution of country keywords in this field to global keywords.

We can classify high-frequency keywords and analyze the strength of relationships between keywords by examining keyword co-occurrence in a large number of literary works and thereby identify the internal structure of an academic field and reveal the research frontiers of the discipline (40). Table 2 lists the 10 most frequent keyword entries and their frequencies, centrality, and first appearance times. Figure 7A shows the co-occurrence network of keywords. This study found that neuroinflammation in ASD was more of a focus in studies on children, especially in the core symptoms of behavioral science; more attention was paid to NF-kappa B, tumor necrosis factor, and microglia on inflammatory cytokines and inflammatory cells. The keyword frequency time distribution heat map in Figure 7B shows that, among the 30 high-frequency keywords, cytokines, oxidative stress, mast cells, and autoimmunity have been of continuous concern. Microglia, astrocytes, intestinal microorganisms, and the gut–brain axis have been the hot spots in recent years. The VOSviewer superimposed visualization in Figures 7C–E show the contribution of the countries whose number of publications ranks among the top three in ASD neuroinflammation. There is no doubt that the US has made outstanding progress in many areas, particularly in the areas of gut microbiology, epigenetics, pathogenesis, and environmental factors; in Italy, there is a similar focus on children and an impressive contribution in genetics and immunology; meanwhile, in Saudi Arabia, there is a major emphasis on oxidative stress, inflammatory dysregulation, and cytokines.

**TABLE 2:** High-frequency keywords.

Ranking	Keyword	Frequency	Centrality	Year of the first appearance
1	Children	131	0.39	2005
2	Gene expression	82	0.22	2005
3	Oxidative stress	78	0.26	2006
4	Activation	74	0.22	2007
5	Microglia	72	0.11	2005
6	Immune response	44	0.11	2005
7	Risk	43	0.06	2006
8	NF kappa b	37	0.08	2014
9	Behavior	33	0.05	2014
10	Brain development	28	0.08	2007
10	Necrosis factor alpha	28	0.05	2010

#### 3.6.2. Clusters of keywords

**FIGURE 8:**
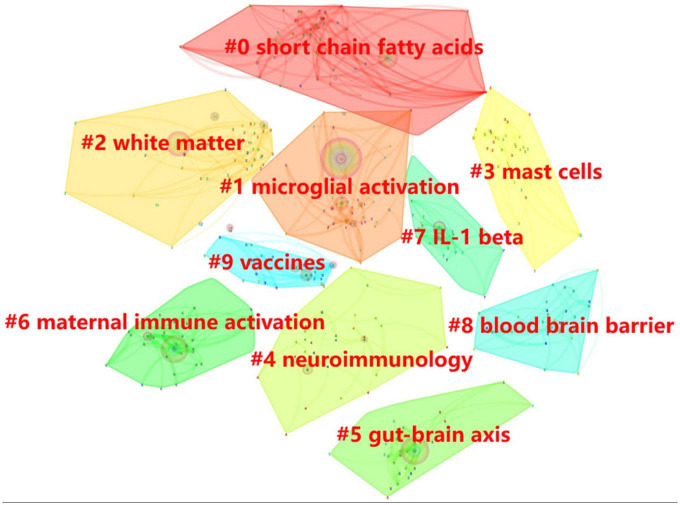
Clusters of keywords: Each color block represents a cluster, and all nodes within the color block belong to the cluster. The larger the cluster number, the smaller the cluster scale.

To understand the basic knowledge structure of the field more thoroughly, we used cluster analysis to categorize data by similarity based on keyword co-occurrence networks (41), obtaining the 10 keyword clusters in Figure 8 in the area of neuroinflammatory disorders in ASD autism. The results of Table 3 show that the silhouette values of the 10 clusters are greater than 0.85, which demonstrates the high homogeneity of the members within the clusters and the high quality of the clustering results. #0 short chain fatty acids and #5 gut-brain axis are classified as intestinal flora; #1microglial activation, #3 mast call, and #7 IL1-β are categorized as cytokines and cells associated with the neuroinflammatory mechanism of ASD; #2 white matter is the brain region of greatest concern in the field; #8 blood brain barrier disorder may be the cause of neuroinflammation; #9 vaccines are classified as risk factors for neuroinflammation of ASD; #4 neuroimmunology and #6 maternal immune activation are grouped into immune pathways.

**TABLE 3:** Details of keyword clusters.

Cluster	Size	Silhouette	Time	Keywords (from highest to lowest word frequency)
Short chain fatty acids	47	0.919	2013	Autism spectrum disorder; pten mutation; mouse model; pm2.5; neuron | oxidative stress; signaling pathway; pm2.5; neuron; magnetic resonance spectroscopy
Microglial activation	39	0.896	2015	Oxidative stress; memory deficits; Alzheimer’s disease; motor dysfunctions; excitatory-inhibitory balance | autism spectrum disorder; event-related potential; memory deficits; excitatory-inhibitory balance; mitochondrial dysfunction
White matter	31	0.934	2012	Autism spectrum disorders; oxidative stress; propanoic acid; nitric oxide pathway; catechin hydrate | systemic lupus; cerebral blood; ecd brain; ischemic penumbra; memory deficits
Mast cells	30	0.929	2010	Autism spectrum disorder; blood-brain barrier; gut microbiota; ketogenic diet; cerebral hypoperfusion | autism; siblings; cytokines; jordan; interleukins
Neuroimmunology	28	0.865	2013	Microglia; drug; beta (2) adrenergic receptor; animal model; brain development | maternal immune activation; autism spectrum disorder; stereotyped behaviors; social preference; communicative deficits
Gut-brain axis	28	0.914	2014	Autism spectrum disorder; gut microbiota; blood-brain barrier; ketogenic diet; attention deficit hyperactivity disorder | gut-brain axis; bacterial metabolites; 4-ethylphenyl sulfate; gulu university; metabolic disorder
Maternal immune activation	19	0.889	2014	Autism spectrum disorder; animal models; autoimmune disorders; maternal immune activation; amyotrophic lateral sclerosis | maternal immune activation; animal models; social cognition; mycobacterium tuberculosis; adrenocorticotropic hormone
IL-1 beta	18	0.878	2017	Oxidative stress; particulate matter; urban environment; vehicular emissions; lymphocyte activation | underconnectivity; spectrum disorder; neuroinflammation; abnormal functional connectivity; microglia
Blood brain barrier	14	0.95	2009	Cell minicolumn; fragile x; spectrum disorder; non-specific coliti; blood serotonin | cannabinoid; pediatrics; neuropsychiatry; neuroinflammation; autism
Vaccines	10	0.973	2015	Autism spectrum disorder; obsessive-compulsive disorder; neurodevelopmental disorders; emotional disturbances; tic disorder | aluminum adjuvants; adjuvant safety; gulf war syndrome; immune response; multiple sclerosis

#### 3.6.3. Keywords bursts

**FIGURE 9:**
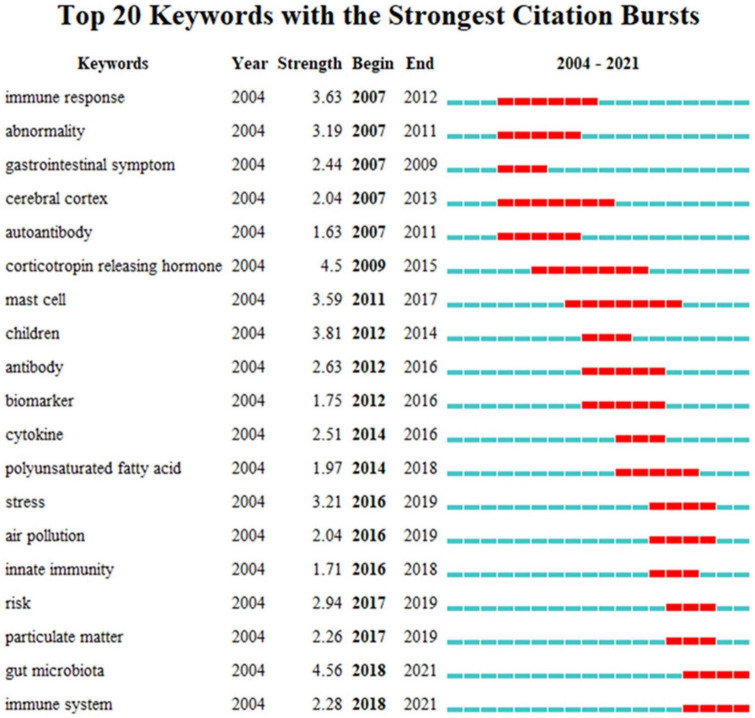
The top 20 keywords with the strongest citation bursts based on CiteSpace. The red horizontal stripes represent the years with the most frequent keyword use. The green horizontal stripes represent the years with the most infrequent keyword use.

Based on the analysis of research hotspots, CiteSpace was applied to identify the burst keywords. The burst map can reflect the incremental increase of keywords in a certain period, according to which the research direction and attention degree of the period are judged (34). The results showed (Figure 9) that between 2007 and 2013, research focused on the cerebral cortex, immune response, adrenocorticotropin-releasing hormone, cerebral cortex, and abnormalities. From 2014 to 2018, mast cells, antibodies, and polyunsaturated fatty acids became hot spots for research on the mechanisms of neuroinflammation in ASD; between 2019 and 2021, the gut microbiota and immune system have drawn sufficient attention to be potential future research trends and frontiers in the field of neuroinflammation in ASD.

### 3.7. The most accumulatively cited 10 experimental papers in the field and most cited 10 experimental papers in the last 2 years

We sorted out the 10 most cited experimental publications cumulatively, but to avoid affecting the citation frequency due to publication time, the 10 most cited experimental publications in the last 2 years (2020 and 2021) were also reviewed. It is useful for researchers to understand the in-depth research in the field of ASD neuroinflammation, as detailed in Tables 4, 5. Of these 20 articles, the most cited were the seminal articles confirming the presence of neuroinflammation in patients with ASD. Overall, not only were specific manifestations of neuroinflammation in ASD highly cited in the past 2 years, but so were maternal immune activation and environmental influences on neuroinflammation in ASD, as well as pharmacological interventions for neuroinflammation. Among the journals, it is worth noting that *Behavioral Brain Research* was the main source of these articles, ranking second in terms of previous journal publications.

**TABLE 4:** The basic information of most accumulatively cited 10 experimental papers in the field.

Ranking	Title	References	Total citations	Result, breakthrough, or major discovery
1	Neuroglial activation and neuroinflammation in the brain of patients with autism	Vargas et al. (7)	1,263	First demonstration of glial cell activation, active neuroinflammation, and abnormal autoimmunity in ASD brain tissue and cerebrospinal fluid.
2	Elevated immune response in the brain of autistic patients	Li et al. (10)	439	ASD patients show increased innate and adaptive immune responses across the Th1 pathway, suggesting that regional brain inflammation and autoimmunity may be involved in the pathogenesis.
3	Neurobiological effects of intraventricular propionic acid in rats: Possible role of short chain fatty acids on the pathogenesis and characteristics of autism spectrum disorders.	Macfabe et al. (54)	270	Altered propionate metabolism is present in some ASD types and ventricular injection of propionate allows for ASD modeling.
4	Evidence of oxidative damage and inflammation associated with low glutathione redox status in the autism brain	Rose et al. (55)	260	Increased markers of oxidative damage and increased oxidative stress in ASD brains suggest a possible chronic inflammatory response, mitochondrial superoxide production, and oxidative protein and DNA damage.
5	Effects of the enteric bacterial metabolic product propionic acid on object-directed behavior, social behavior, cognition, and neuroinflammation in adolescent rats: Relevance to autism spectrum disorder.	Macfabe et al. (56)	216	ASD-relevant behavioral and intrinsic neuroinflammatory response with astrocyte proliferation and microglia activation in adult rats given PPA.
6	Cerebrospinal fluid and serum markers of inflammation in autism	Zimmerman et al. (57)	211	Metabolic pathways and absence of concurrent infection, respectively, in autism. Alternatively, they may be produced by microglia but remain localized and not expressed in cerebrospinal fluid.
7	Memantine as adjunctive therapy in children diagnosed with autistic spectrum disorders: An observation of initial clinical response and maintenance tolerability.	Chez (58)	154	Language function, social behavior, and self-stimulatory behaviors, although self-stimulatory behaviors comparatively improved to a lesser degree after using memantine in ASD patients.
8	Neurotoxicity of traffic-related air pollution.	Costa et al. (59)	149	The most prominent effects caused by air pollution in both humans and animals are oxidative stress and neuro-inflammation.
9	Blood-brain barrier and intestinal epithelial barrier alterations in autism spectrum disorders	Fiorentino et al. (12)	147	In the ASD brain, there is an altered expression of genes associated with BBB integrity coupled with increased neuroinflammation and possibly impaired gut barrier integrity.
10	Altered gut microbiota and short chain fatty acids in Chinese children with autism spectrum disorder	Liu et al. (60)		ASD subjects have lower levels of fecal acetic acid and butyrate and a higher level of fecal valeric acid, and decreased abundances of key butyrate-producing taxa and an increased abundance of valeric acid associated bacteria among autistic individuals.

**TABLE 5:** The basic information of the most cited 10 experimental papers in the last 2 years.

Ranking	Title	References	Total citations	Result, breakthrough, or major discovery
1	Prenatal stress causes intrauterine inflammation and serotonergic dysfunction, and long-term behavioral deficits through microbe- and CCL2-dependent mechanisms.	Chen et al. (61)	22	A complex interaction between maternal microbes, inflammation, and serotonin metabolism regulates the emergence of behavioral abnormalities following Prenatal stress.
2	Infusion of human umbilical cord tissue mesenchymal stromal cells in children with autism spectrum disorder.	Sun et al. (62)	19	A small phase I trial of human umbilical tissue mesenchymal stromal cells appear to be safe and feasible in young children with ASD.
3	Prenatal exposure to bisphenol A alters the transcriptome-interactome profiles of genes associated with Alzheimer’s disease in the offspring hippocampus	Sukjamnong et al. (63)	18	Maternal prenatal exposure to bisphenol A exposure may increase AD risk in offspring by dysregulating genes associated with AD neuropathology and inflammation and reveal a possible relationship between AD and autism
4	Adenylate cyclase activator forskolin alleviates intracerebroventricular propionic acid-induced mitochondrial dysfunction of autistic rats	Mehan et al. (64)	18	Forskolin can alleviate neuronal mitochondrial dysfunction and improve neurological symptoms of rats with autism.
5	[^11^C]P PBR28 MR-PET imaging reveals lower regional brain expression of translocator protein (TSPO) in young adult males with autism spectrum disorder	Zurcher et al. (65)	15	Young adult males with ASD exhibited lower regional TSPO expression in several brain regions, including the bilateral insular cortex, bilateral precuneus/posterior cingulate cortex, and bilateral temporal, angular, and supramarginal gyri.
5	Increased extracellular free-water in adult male rats following *in utero* exposure to maternal immune activation	Di Biase et al. (66)	15	Excess free water across frontal white matter fibers of rats exposed to prenatal immune activation
7	Maternal immune activation induces neuroinflammation and cortical synaptic deficits in the adolescent rat offspring	Cieslik et al. (67)	14	Long-term changes in synaptic structure and protein levels caused by maternal immune activation may lead to behavioral abnormalities associated with autism and related disorders in offspring
8	Maternal autoimmunity and inflammation are associated with childhood tics and obsessive-compulsive disorder: Transcriptomic data show common enriched innate immune pathways.	Jones et al. (68)	13	Maternal pro-inflammatory status is associated with childhood tic disorder/OCD and supports a possible role for maternal inflammation in the etiology of tic disorder and OCD beyond immunogenetic and “neurogenic” mechanisms.
9	Neuroprotective effect of alpha-mangostin in ameliorating propionic acid-induced experimental model of autism in Wistar rats	Tiwari et al. (69)	13	Alpha-Mangostin reduces excessive activation of extracellular signal-regulated protein kinase signaling and restores autistic-like behavioral and neurochemical changes.
10	MEF2C hypofunction in neuronal and neuroimmune populations produces MEF2C haploinsufficiency syndrome-like behaviors in mice.	Harrington et al. (70)	12	Myocyte Enhancer Factor 2 regulates typical brain development and function through multiple cell types, including excitatory neurons and neuroimmune populations.

## 4. Discussion

This study used a bibliometric approach to analyze the development of neuroinflammation research in ASD over the past 18 years. The quantitative explosion of documentation in this area between 2004 and 2021, particularly after 2013, may be a result of increasing recognition of the role of neuroinflammation in the development of ASD and therefore increased funding for research in this area. In terms of the type of literature, the number of review papers has exceeded half of the experimental papers. On the one hand, the research on neuroinflammation in ASD is in the preliminary exploration stage, and there is relatively little relevant original research. On the other hand, the research methodology in this field is complex and has high demands on the relevant subject groups. Nevertheless, the multiplicity of review articles indicates that researchers maintain a high level of interest in the field, suggesting the importance of neuroinflammation in ASD-related research, which deserves more attention in the future. In terms of countries/regions, the majority of articles are from the United States, Italy, Saudi Arabia, China, and England, which is strongly correlated with the economic development of the countries (42). In addition, government spending on health care may be an essential factor. With the United States having the highest health care expenditures in the world (43, 44), it makes sense that the country has the highest number of publications and concentration of collaborations. Three of the top five institutions in terms of number of publications belong to the US, so the US is the largest contributor in this field. Among the top 10 journals in terms of publication volume, publishers are centralized in Europe and the United States, although Saudi Arabia and China are also two key countries in the field of neuroinflammation in ASD, there are no publishers in Asia, which highlights the significance of developing influential international journals in Asia. In terms of author collaboration, it is worth noting that Laila Al-Ayadhi, connects three different clusters, one possibly benefiting from the advantage of being in the same country and the other possibly thanks to the RUDN University Project 5–100 program in Russia, which connects it to authors from other countries.

From the keyword co-occurrence and clustering results of neuroinflammation in ASD research, 491 subject words were clustered into 10 research topics, which are both independent of each other and accompany each other, forming an intricate network structure. From these, this review summarizes four institutional pathways that have been the focus of attention in recent years: (1) Microglia-mediated. Microglia activation in the central nervous system in ASD has been of persistent interest since its identification in 2005. The prevailing view is that microglia subtype dysregulation is strongly involved in the occurrence and development of neuroinflammation in ASD. In patients with ASD, microglia are abnormally activated, there are phenotypic imbalances, and a large number of inflammatory mediators are released, resulting in excessive and persistent immune disharmony, destroying neuronal structure and function, and even engulfing synapses and entire neurons, contributing to abnormal brain development and immature connections (45). (2) Mast cell-mediated. Since the clinical finding in 2011 that ASD is characterized by a tenfold increase in the incidence of mastocytosis compared with the normal population (46), attention has turned to the role of mast cells in neuroinflammation and ASD. It is currently recognized that neuroinflammation is affected by two main pathways: the interaction of mast cells with glial cells and neurons, leading to the release of mediators such as cytokines, protein hydrolases, and reactive oxygen species (47), and the direct release of mediators such as tumor necrosis factor-α, histamine, and lactase from mast cells, ultimately affecting neurogenesis, neurodegeneration, and the permeability of the blood-brain barrier. (3) Tumor necrosis factor-α (TNF-α)-mediated. Visualization results demonstrate that TNF-α is the cytokine that has received the most attention in studies related to neuroinflammation in ASD. It was found that high levels of TNF-α in the serum and brain of ASD patients were positively correlated with the severity of symptoms in autistic patients and that the expression of THRIL, a related gene that regulates TNF-α, was decreased (48). In addition to the possible pathogenesis of ASD, in the peripheral system, TNF-α enters the brain from the peripheral blood and directly affects brain function through its receptors (49); in the central nervous system, microglia and mast cells can directly secrete TNF-α, which drives the immune inflammatory response in the central nervous system. (4) Nuclear factor kappa-B (NF-kB)-mediated. NF-kB consists of a series of transcription factors that are present in almost all cells and is the major regulator of inflammation and immune homeostasis, playing a critical role in many inflammatory diseases (50). Ever since the discovery of significantly elevated NF-kB expression in the peripheral blood of ASD patients (51) and a significant increase in NF-kB expression in the brain of ASD mice, researchers have targeted NF-KB from 2014 onward. Current studies suggest that NF-kB is a convergence of multiple pathways in the pathogenesis of ASD, such as high expression of interleukin-17 receptor A potentially increasing the expression of NF-kB through activating the NF-kB pathway (21) or Toll-like receptor signal transduction (19). NF-kB is activated in response to these stressor stimuli, which drives the overexpression of pro-inflammatory genes (including cytokines, chemokines, and adhesion molecule expression), and plenty of pro-inflammatory factors are produced, including IL-1 and TNF-α, which in turn act as activators of NF-kB, creating a complex positive feedback that keeps NF-kB in a hyper-activated state, subsequently leading to a vicious cycle of excessive and uncontrolled neuroinflammation (52). Keyword burst is an essential means of investigating the evolution of academic research hotspots, and the intestinal microbiota and immune system have been continuous bursting keywords since 2019, which is consistent with the development of the microbiome-gut-brain axis hypothesis in the field of ASD neuroinflammation, and the intestinal microflora regulates changes in immunity and inflammation through the gut–brain axis, influencing the occurrence and development of ASD (53). Therefore, targeting specific gut microbiota and immunity may be the future direction of ASD treatment.

This study also has a few limitations. First, the research was limited to the use of the WOS database as a source of data collection; subsequent studies can include more academic databases (such as PubMed, Scope, and Google Scholar) so that the research findings are more objective and comprehensive. Second, this paper only analyzed the co-citation and co-occurrence diagrams, and future investigations using bibliometric-coupled methods will help deepen researchers’ understanding of the trends of neuroinflammation in ASD.

## 5. Conclusion

To sum up, this study is the first to use quantitative methods to analyze the research history and development status of ASD neuroinflammation and to visualize the number of publications, countries, journals, authors, institutions, and keywords in the field of ASD neuroinflammation. We found that the annual number of publications has skyrocketed in recent years, and people are paying more and more attention to ASD neuroinflammation, with the United States being the country with the largest contribution and the *Brain Behavior and Immunity* being the journal with the most publications and citations. We also identified the key individuals and institutions involved in researching this field and summarized the current hotspot mechanisms of concern. We expect that these findings will provide a new research direction in the mechanism of inflammation and lay a foundation for further research on the development trend and focus of neuroinflammation, the establishment of academic exchanges and cooperation between different academic groups, and the promotion of in-depth research on ASD neuroinflammation. In future research, we can seek to investigate the related pathways, including cytokine and glial cell changes in neuroinflammation in ASD, and immunotherapy for inflammation can be used as a possible direction for ASD intervention programs to promote the recovery of ASD patients.

## Data availability statement

The original contributions presented in this study are included in this article/Supplementary material, further inquiries can be directed to the corresponding author.

## Author contributions

YS, J-GZ, and X-HH: study design. YS, J-GZ, W-TL, Y-HL, J-HG, and B-XZ: data collection, analysis, and interpretation. YS: drafting of the manuscript. X-HH: critical revision of the manuscript. All authors contributed to the article and approved the submitted version.

## References

[1] Lobar SL. DSM-V changes for autism spectrum disorder (ASD): implications for diagnosis, management, and care coordination for children with ASDs. *J Pediatr Health Care.* (2016) 30:359–65. 10.1016/j.pedhc.2015.09.005 26602109

[2] Baio J Wiggins L Christensen DL Maenner MJ Daniels J Warren Z Prevalence of autism spectrum disorder among children aged 8 years - autism and developmental disabilities monitoring network, 11 sites, United States, 2014. *MMWR Surveill Summ.* (2018) 67:1–23. 10.15585/mmwr.ss6706a1 29701730 PMC5919599

[3] Lyall K Croen L Daniels J Fallin MD Ladd-Acosta C Lee BK The changing epidemiology of autism spectrum disorders. *Annu Rev Public Health.* (2017) 38:81–102. 10.1146/annurev-publhealth-031816-044318 28068486 PMC6566093

[4] Manoli DS State MW. Autism spectrum disorder genetics and the search for pathological mechanisms. *Am J Psychiatry.* (2021) 178:30–8. 10.1176/appi.ajp.2020.20111608 33384012 PMC8163016

[5] Pretzsch CM Schäfer T Lombardo MV Warrier V Mann C Bletsch A Neurobiological correlates of change in adaptive behavior in autism. *Am J Psychiatry.* (2022) 179:336–49. 10.1176/appi.ajp.21070711 35331004

[6] Satterstrom FK Kosmicki JA Wang J Breen MS De Rubeis S An JY Large-scale exome sequencing study implicates both developmental and functional changes in the neurobiology of autism. *Cell.* (2020) 180:568–84.e23. 10.1016/j.cell.2019.12.036 31981491 PMC7250485

[7] Vargas DL Nascimbene C Krishnan C Zimmerman AW Pardo CA. Neuroglial activation and neuroinflammation in the brain of patients with autism. *Ann Neurol.* (2005) 57:67–81. 10.1002/ana.20315 15546155

[8] Liao X Liu Y Fu X Li Y. Postmortem studies of neuroinflammation in autism spectrum disorder: a systematic review. *Mol Neurobiol.* (2020) 57:3424–38. 10.1007/s12035-020-01976-5 32529489

[9] Han VX Patel S Jones HF Dale RC. Maternal immune activation and neuroinflammation in human neurodevelopmental disorders. *Nat Rev Neurol.* (2021) 17:564–79. 10.1038/s41582-021-00530-8 34341569

[10] Li XH Chauhan A Sheikh AM Patil S Chauhan V Li XM Elevated immune response in the brain of autistic patients. *J Neuroimmunol.* (2009) 207:111–6. 10.1016/j.jneuroim.2008.12.002 19157572 PMC2770268

[11] Suzuki K Sugihara G Ouchi Y Nakamura K Futatsubashi M Takebayashi K Microglial activation in young adults with autism spectrum disorder. *Jama Psychiatry.* (2013) 70:49–58. 10.1001/jamapsychiatry.2013.272 23404112

[12] Fiorentino M Sapone A Senger S Camhi SS Kadzielski SM Buie TM Blood-brain barrier and intestinal epithelial barrier alterations in autism spectrum disorders. *Mol Autism.* (2016) 7:49. 10.1186/s13229-016-0110-z 27957319 PMC5129651

[13] Sciara AN Beasley B Crawford JD Anderson EP Carrasco T Zheng SM Neuroinflammatory gene expression alterations in anterior cingulate cortical white and gray matter of males with autism spectrum disorder. *Autism Res.* (2020) 13:870–84. 10.1002/aur.2284 32129578 PMC7540672

[14] Vakilzadeh G Falcone C Dufour B Hong T Noctor SC Martinez-Cerdeno V. Decreased number and increased activation state of astrocytes in gray and white matter of the prefrontal cortex in autism. *Cereb Cortex.* (2022) 32:4902–12. 10.1093/cercor/bhab523 35212358 PMC9627019

[15] Tsilioni I Patel AB Pantazopoulos H Berretta S Conti P Leeman SE IL-37 is increased in brains of children with autism spectrum disorder and inhibits human microglia stimulated by neurotensin. *Proc Natl Acad Sci USA.* (2019) 116:21659–65. 10.1073/pnas.1906817116 31591201 PMC6815178

[16] Menassa DA Sloan C Chance SA. Primary olfactory cortex in autism and epilepsy: increased glial cells in autism. *Brain Pathol.* (2017) 27:437–48. 10.1111/bpa.12415 27409070 PMC8029489

[17] Lee AS Azmitia EC Whitaker-Azmitia PM. Developmental microglial priming in postmortem autism spectrum disorder temporal cortex. *Brain Behav Immun.* (2017) 62:193–202. 10.1016/j.bbi.2017.01.019 28159644

[18] Nadeem A Ahmad SF Al-Harbi NO Al-Ayadhi LY Sarawi W Attia SM Imbalance in pro-inflammatory and anti-inflammatory cytokines milieu in B cells of children with autism. *Mol Immunol.* (2022) 141:297–304. 10.1016/j.molimm.2021.12.009 34915269

[19] Nadeem A Ahmad SF Attia SM Al-Ayadhi LY Bakheet SA Al-Harbi NO. Oxidative and inflammatory mediators are upregulated in neutrophils of autistic children: role of IL-17A receptor signaling. *Prog Neuropsychopharmacol Biol Psychiatry.* (2019) 90:204–11. 10.1016/j.pnpbp.2018.12.002 30529000

[20] Nadeem A Ahmad SF Attia SM Al-Ayadhi LY Al-Harbi NO Bakheet SA. Dysregulation in IL-6 receptors is associated with upregulated IL-17A related signaling in CD4+ T cells of children with autism. *Prog Neuropsychopharmacol Biol Psychiatry.* (2020) 97:109783. 10.1016/j.pnpbp.2019.109783 31655158

[21] Nadeem A Ahmad SF Attia SM Bakheet SA Al-Harbi NO Al-Ayadhi LY. Activation of IL-17 receptor leads to increased oxidative inflammation in peripheral monocytes of autistic children. *Brain Behav Immun.* (2018) 67:335–44. 10.1016/j.bbi.2017.09.010 28935156

[22] Glanzel W Czerwon HJA. new methodological approach to bibliographic coupling and its application to the national, regional and institutional level. *Scientometrics.* (1996) 37:195–221.

[23] Glänzel W Czerwon HJ. A new methodological approach to bibliographic coupling and its application to the national regional and institutional level. *Scientometrics.* (1996) 37:195–221.

[24] Du L Luo S Liu G Wang H Zheng L Zhang Y. The 100 top-cited studies about pain and depression. *Front Psychol.* (2019) 10:3072. 10.3389/fpsyg.2019.03072 32116876 PMC7026489

[25] Brandt JS Hadaya O Schuster M Rosen T Sauer MV Ananth CVA. Bibliometric analysis of top-cited journal articles in obstetrics and gynecology. *JAMA Netw Open.* (2019) 2:e1918007. 10.1001/jamanetworkopen.2019.18007 31860106 PMC6991228

[26] Hirsch JE. An index to quantify an individual’s scientific research output. *Proc Natl Acad Sci USA.* (2005) 102:16569–72. 10.1073/pnas.0507655102 16275915 PMC1283832

[27] Sun HL Bai W Li XH Huang HH Cui XL Cheung T Schizophrenia and inflammation research: a bibliometric analysis. *Front Immunol.* (2022) 13:907851. 10.3389/fimmu.2022.907851 35757702 PMC9219580

[28] Wu H Zhou Y Wang Y Tong L Wang F Song S Current state and future directions of intranasal delivery route for central nervous system disorders: a scientometric and visualization analysis. *Front Pharmacol.* (2021) 12:717192. 10.3389/fphar.2021.717192 34322030 PMC8311521

[29] Van Eck NJ Waltman L. Software survey: VOSviewer, a computer program for bibliometric mapping. *Scientometrics.* (2010) 84:523–38. 10.1007/s11192-009-0146-3 20585380 PMC2883932

[30] Chen C. *Citespace: a practical guide for mapping scientific literature.* New York, NY: Nova Science Publishers (2016).

[31] Darvish H. Bibliometric analysis using bibliometrix an r package. *J Sci Res.* (2020) 8:156–60. 10.5530/jscires.8.3.32

[32] Eck NJ Waltman L. Visualizing bibliometric networks. In: Ding Y Rousseau R Wolfram D editors. *Measuring scholarly impact.* Cham: Springer (2014). p. 285–320.

[33] Fan JC Gao Y Zhao N Dai RJ Zhang HL Feng XY Bibliometric analysis on covid-19: a comparison of research between English and Chinese studies. *Front Public Health.* (2020) 8:477. 10.3389/fpubh.2020.00477 32923422 PMC7456831

[34] Chen CM. CiteSpace II: detecting and visualizing emerging trends and transient patterns in scientific literature. *J Am Soc Inform Sci Technol.* (2006) 57:359–77. 10.1002/asi.20317

[35] You Y Wang D Wang Y Li Z Ma XA. Bird’s-eye view of exercise intervention in treating depression among teenagers in the last 20 years: a bibliometric study and visualization analysis. *Front Psychiatry.* (2021) 12:661108. 10.3389/fpsyt.2021.661108 34220574 PMC8249759

[36] Qiang R Zhao Z Tang L Wang Q Wang Y Huang Q. Identification of 5 hub genes related to the early diagnosis, tumour stage, and poor outcomes of hepatitis b virus-related hepatocellular carcinoma by bioinformatics analysis. *Comput Math Methods Med.* (2021) 2021:9991255. 10.1155/2021/9991255 34603487 PMC8483908

[37] Chen Y Lin M Zhuang D. Wastewater treatment and emerging contaminants: bibliometric analysis. *Chemosphere.* (2022) 297:133932. 10.1016/j.chemosphere.2022.133932 35149018

[38] Abu-Odah H Molassiotis A Liu JY. Global palliative care research (2002-2020): bibliometric review and mapping analysis. *BMJ Support Palliat Care.* (2021) 12:376–87. 10.1136/bmjspcare-2021-002982 34373283 PMC9691821

[39] Yao RQ Ren C Wang JN Wu GS Zhu XM Xia ZF Publication trends of research on sepsis and host immune response during 1999-2019: a 20-year bibliometric analysis. *Int J Biol Sci.* (2020) 16:27–37. 10.7150/ijbs.37496 31892843 PMC6930382

[40] Radhakrishnan S Erbis S Isaacs JA Kamarthi S. Novel keyword co-occurrence network-based methods to foster systematic reviews of scientific literature. *PLoS One.* (2017) 12:e0172778. 10.1371/journal.pone.0172778 28328983 PMC5362196

[41] Chen CM Ibekwe-Sanjuan F Hou JH. The structure and dynamics of cocitation clusters: a multiple-perspective cocitation analysis. *J Am Soc Inform Sci Technol.* (2010) 61:1386–409. 10.1002/asi.21309

[42] Kiraz M Demir E. A bibliometric analysis of publications on spinal cord injury during 1980–2018. *World Neurosurg.* (2020) 136:e504–13. 10.1016/j.wneu.2020.01.064 31954906

[43] Wang L Leslie DL. Health care expenditures for children with autism spectrum disorders in medicaid. *J Am Acad Child Adolesc Psychiatry.* (2010) 49:1165–71. 10.1016/j.jaac.2010.08.003 20970704 PMC3047439

[44] World Health Organization [WHO]. *Global health expenditure database[EB/OL].* (2020). Available online at: https://apps.who.int/nha/database/Country_Profile/Index/en (accessed September 29, 2022).

[45] Matta SM Hill-Yardin EL Crack PJ. The influence of neuroinflammation in autism spectrum disorder. *Brain Behav Immun.* (2019) 79:75–90. 10.1016/j.bbi.2019.04.037 31029798

[46] Angelidou A Alysandratos KD Asadi S Zhang B Francis K Vasiadi M Brief report: “allergic symptoms” in children with autism spectrum disorders. More than meets the eye? *J Autism Dev Disord.* (2011) 41:1579–85. 10.1007/s10803-010-1171-z 21210299

[47] Theoharides TC Stewart JM Panagiotidou S Melamed I. Mast cells, brain inflammation and autism. *Eur J Pharmacol.* (2016) 778:96–102. 10.1016/j.ejphar.2015.03.086 25941080

[48] Xie J Huang L Li X Li H Zhou Y Zhu H Immunological cytokine profiling identifies TNF-α as a key molecule dysregulated in autistic children. *Oncotarget.* (2017) 8:82390–8. 10.18632/oncotarget.19326 29137272 PMC5669898

[49] Banks WA. The blood-brain barrier in neuroimmunology: tales of separation and assimilation. *Brain Behav Immun.* (2015) 44:1–8. 10.1016/j.bbi.2014.08.007 25172555 PMC4275374

[50] Mitchell JP Carmody RJNF. -κB and the transcriptional control of inflammation. *Int Rev Cell Mol Biol.* (2018) 335:41–84. 10.1016/bs.ircmb.2017.07.007 29305014

[51] Qasem H Al-Ayadhi L Bjørklund G Chirumbolo S El-Ansary A. Impaired lipid metabolism markers to assess the risk of neuroinflammation in autism spectrum disorder. *Metab Brain Dis.* (2018) 33:1141–53. 10.1007/s11011-018-0206-6 29569150

[52] Zheng S Wang C Lin L Mu S Liu H Hu X TNF-α impairs pericyte-mediated cerebral microcirculation via the nf-κb/inos axis after experimental traumatic brain injury. *J Neurotrauma.* (2022). 10.1089/neu.2022.0016 [Epub ahead of print]. 35972751

[53] Sharon G Cruz NJ Kang DW Gandal MJ Wang B Kim YM Human gut microbiota from autism spectrum disorder promote behavioral symptoms in mice. *Cell.* (2019) 177:1600–18.e17. 10.1016/j.cell.2019.05.004 31150625 PMC6993574

[54] Macfabe DF Cain DP Rodriguez-Capote K Franklin AE Hoffman JE Boon F Neurobiological effects of intraventricular propionic acid in rats: possible role of short chain fatty acids on the pathogenesis and characteristics of autism spectrum disorders. *Behav Brain Res.* (2007) 176:149–69. 10.1016/j.bbr.2006.07.025 16950524

[55] Rose S Melnyk S Pavliv O Bai S Nick TG Frye RE Evidence of oxidative damage and inflammation associated with low glutathione redox status in the autism brain. *Trans Psychiatry.* (2012) 2:e134. 10.1038/tp.2012.61 22781167 PMC3410618

[56] Macfabe DF Cain NE Boon F Ossenkopp KP Cain DP. Effects of the enteric bacterial metabolic product propionic acid on object-directed behavior, social behavior, cognition, and neuroinflammation in adolescent rats: relevance to autism spectrum disorder. *Behav Brain Res.* (2011) 217:47–54. 10.1016/j.bbr.2010.10.005 20937326

[57] Zimmerman AW Jyonouchi H Comi AM Connors SL Milstien S Varsou A Cerebrospinal fluid and serum markers of inflammation in autism. *Pediatr Neurol.* (2005) 33:195–201. 10.1016/j.pediatrneurol.2005.03.014 16139734

[58] Chez MG Burton Q Dowling T Chang MN Khanna P Kramer C. Memantine as adjunctive therapy in children diagnosed with autistic spectrum disorders: an observation of initial clinical response and maintenance tolerability. *J Child Neurol.* (2007) 22:574–9. 10.1177/0883073807302611 17690064

[59] Costa LG Cole TB Coburn J Chang Y Dao K Roque PJ. Neurotoxicity of traffic-related air pollution. *Neurotoxicology.* (2017) 59:133–9. 10.1016/j.neuro.2015.11.008 26610921 PMC4875879

[60] Liu SM Li EY Sun ZY Fu DJ Duan GQ Jiang MM Altered gut microbiota and short chain fatty acids in Chinese children with autism spectrum disorder. *Sci Rep.* (2019) 9:287. 10.1038/s41598-018-36430-z 30670726 PMC6342986

[61] Chen HJ Antonson AM Rajasekera TA Patterson JM Bailey MT Gur TL. Prenatal stress causes intrauterine inflammation and serotonergic dysfunction, and long-term behavioral deficits through microbe- and CCL2-dependent mechanisms. *Trans Psychiatry.* (2020) 10:191. 10.1038/s41398-020-00876-5 32546752 PMC7297973

[62] Sun JS Dawson G Franz L Howard J Mclaughlin C Kistler B Infusion of human umbilical cord tissue mesenchymal stromal cells in children with autism spectrum disorder. *Stem Cells Trans Med.* (2020) 9:1137–46. 10.1002/sctm.19-0434 32531111 PMC7519773

[63] Sukjamnong S Thongkorn S Kanlayaprasit S Saeliw T Hussem K Warayanon W Prenatal exposure to bisphenol A alters the transcriptome-interactome profiles of genes associated with Alzheimer’s disease in the offspring hippocampus. *Sci Rep.* (2020) 10:9487. 10.1038/s41598-020-65229-0 32528016 PMC7289845

[64] Mehan S Rahi S Tiwari A Kapoor T Rajdev K Sharma R Adenylate cyclase activator forskolin alleviates intracerebroventricular propionic acid-induced mitochondrial dysfunction of autistic rats. *Neural Regenerat Res.* (2020) 15:1140–9. 10.4103/1673-5374.270316 31823895 PMC7034277

[65] Zurcher NR Loggia ML Mullett JE Tseng C Bhanot A Richey L C-11 PBR28 MR-PET imaging reveals lower regional brain expression of translocator protein (TSPO) in young adult males with autism spectrum disorder. *Mol Psychiatry.* (2021) 26:1659–69. 10.1038/s41380-020-0682-z 32076115 PMC8159742

[66] Di Biase MA Katabi G Piontkewitz Y Cetin-Karayumak S Weiner I Pasternak O. Increased extracellular free-water in adult male rats following in utero exposure to maternal immune activation. *Brain Behav Immun.* (2020) 83:283–7. 10.1016/j.bbi.2019.09.010 31521731

[67] Cieslik M Gassowska-Dobrowolska M Jesko H Czapski GA Wilkaniec A Zawadzka A Maternal immune activation induces neuroinflammation and cortical synaptic deficits in the adolescent rat offspring. *Int J Mol Sci.* (2020) 21:4097. 10.3390/ijms21114097 32521803 PMC7312084

[68] Jones HF Han VX Patel S Gloss BS Soler N Ho A Maternal autoimmunity and inflammation are associated with childhood tics and obsessive-compulsive disorder: transcriptomic data show common enriched innate immune pathways. *Brain Behav Immun.* (2021) 94:308–17. 10.1016/j.bbi.2020.12.035 33422639

[69] Tiwari A Khera R Rahi S Mehan S Makeen HA Khormi YH Neuroprotective effect of alpha-mangostin in ameliorating propionic acid-induced experimental model of autism in wistar rats. *Brain Sci.* (2021) 11:288. 10.3390/brainsci11030288 33669120 PMC7996534

[70] Harrington AJ Bridges CM Berto S Blankenship K Cho JN Assali A MEF2C hypofunction in neuronal and neuroimmune populations produces mef2c haploinsufficiency syndrome-like behaviors in mice. *Biol Psychiatry.* (2020) 88:488–99. 10.1016/j.biopsych.2020.03.011 32418612 PMC7483399

